# Rinderpest and Peste des Petits Ruminants: Virus Plagues of Large and Small Ruminants

**DOI:** 10.3201/eid1612.100923

**Published:** 2010-12

**Authors:** Muhammad Munir

**Affiliations:** Author affiliation: Swedish University of Agricultural Sciences, Uppsala, Sweden

**Keywords:** Rinderpest, peste des petits ruminants, RP, PPR, virus, disease, control, book review

In Rinderpest and Peste des Petits Ruminants: Virus Plagues of Large and Small Ruminants, Tom Barrett (now deceased), of the Institute for Animal Health, Pirbright Laboratory, Surrey, UK, and his co-editors have subtly presented the main developments in the quest to conquer these diseases. The instructive text, which touches on the key dynamics of both deadly diseases, incorporates considerable historical detail, infection biology, and information on disease diagnosis, control, and eradication. This book consists of high quality scientific and historical research based on the editors’ experience with morbilliviruses and collaborations with other researchers worldwide. In total, 22 scientists have contributed their expertise on various infectious diseases to the monograph’s 17 chapters.

Throughout, the contributors have tried to maintain an appropriate balance between peste des petits ruminants (PPR) and rinderpest (RP). This hypothesis-based balancing act is important to understand PPR. A future significance of PPR can be realized by the phrase stated by the editors, “If rinderpest becomes a disease of the past, PPR is certainly a disease of the future.” The book starts with an historical account of the RP and PPR diseases accompanied by photographs from the 18th century. These photographs are detailed, illustrative, and fascinating. Of particular interest are a condolence letter, written by Emile Roux from Institute Pasteur to the widow of Joseph Hamoir, with whom Emile Roux worked on rinderpest, and a group photo, including Robert Koch, of his visit to the Imperial Veterinary Laboratory (currently Indian Veterinary Research Institute, Mukteshwar, India) in 1897 where he conducted experiments to immunize cattle with the bile taken from an animal that had succumbed in a virulent outbreak of rinderpest. 

The book focuses on the following issues: relative position of each member in the genera, comparative molecular biology, pathophysiology of the infectious diseases, global epidemiologic patterns, contribution of countries in the eradication of the disease under the Pan African Rinderpest Campaign Programme and the Pan African Programme for the Control of Epizootics, viral immune suppression, and molecular diagnostic approaches being developed. For all these issues, the importance for clinicians of accurate diagnosis and management and prevention of infectious diseases is highlighted. Because of the current sensitivity about an emergence of PPR and the successful Global Rinderpest Eradication Programme, the contributors believe that PPR can be controlled similarly and its spread prevented. Thus, the last 7 chapters emphasize the traditional prophylactic measures, potency of vaccines and possibility of vaccine use, the history of vaccine improvement, recent advances in vaccine development, implementation of international control campaigns for the eradication of RP and PPR by using vaccine, and a brief overview of the pathogenesis and eradication of measles virus. Finally, the editors assess the real cost and benefits of the Global Rinderpest Eradication Programme campaign and predict that the world will soon be free of RP, at comparatively little cost.

Although the book discusses many aspects of the current situation, such as epidemiological distribution, progress in vaccine development, and advances in the diagnosis of PPR diagnostic procedures, information regarding the most recent developments is lacking; a few chapters were written in ≈2000 before the increase in PPR research. Still, I consider this to be the only book that comprehensively describes PPR. It is suitable not only for academics and researchers, but also for virologists, infectious disease specialists, vaccine researchers, and clinicians. Moreover, this book provides appropriate scientific source material suitable for undergraduate and graduate studies. 

